# Superior protein thermophilicity prediction with protein language model embeddings

**DOI:** 10.1093/nargab/lqad087

**Published:** 2023-10-11

**Authors:** Florian Haselbeck, Maura John, Yuqi Zhang, Jonathan Pirnay, Juan Pablo Fuenzalida-Werner, Rubén D Costa, Dominik G Grimm

**Affiliations:** Technical University of Munich, Campus Straubing for Biotechnology and Sustainability, Bioinformatics, 94315 Straubing, Germany; Weihenstephan-Triesdorf University of Applied Sciences, Bioinformatics, 94315 Straubing, Germany; Technical University of Munich, Campus Straubing for Biotechnology and Sustainability, Bioinformatics, 94315 Straubing, Germany; Weihenstephan-Triesdorf University of Applied Sciences, Bioinformatics, 94315 Straubing, Germany; Technical University of Munich, Campus Straubing for Biotechnology and Sustainability, Bioinformatics, 94315 Straubing, Germany; Technical University of Munich, Campus Straubing for Biotechnology and Sustainability, Bioinformatics, 94315 Straubing, Germany; Weihenstephan-Triesdorf University of Applied Sciences, Bioinformatics, 94315 Straubing, Germany; Technical University of Munich, Campus Straubing for Biotechnology and Sustainability, Chair of Biogenic Functional Materials, 94315 Straubing, Germany; Technical University of Munich, Campus Straubing for Biotechnology and Sustainability, Chair of Biogenic Functional Materials, 94315 Straubing, Germany; Technical University of Munich, Campus Straubing for Biotechnology and Sustainability, Bioinformatics, 94315 Straubing, Germany; Weihenstephan-Triesdorf University of Applied Sciences, Bioinformatics, 94315 Straubing, Germany; Technical University of Munich, TUM School of Computation, Information and Technology (CIT), 85748 Garching, Germany

## Abstract

Protein thermostability is important in many areas of biotechnology, including enzyme engineering and protein-hybrid optoelectronics. Ever-growing protein databases and information on stability at different temperatures allow the training of machine learning models to predict whether proteins are thermophilic. *In silico* predictions could reduce costs and accelerate the development process by guiding researchers to more promising candidates. Existing models for predicting protein thermophilicity rely mainly on features derived from physicochemical properties. Recently, modern protein language models that directly use sequence information have demonstrated superior performance in several tasks. In this study, we evaluate the usefulness of protein language model embeddings for thermophilicity prediction with ProLaTherm, a **Pro**tein **La**nguage model-based **Therm**ophilicity predictor. ProLaTherm significantly outperforms all feature-, sequence- and literature-based comparison partners on multiple evaluation metrics. In terms of the Matthew’s correlation coefficient, ProLaTherm outperforms the second-best competitor by 18.1% in a nested cross-validation setup. Using proteins from species not overlapping with species from the training data, ProLaTherm outperforms all competitors by at least 9.7%. On these data, it misclassified only one nonthermophilic protein as thermophilic. Furthermore, it correctly identified 97.4% of all thermophilic proteins in our test set with an optimal growth temperature above 70°C.

## Introduction

The thermostability of proteins is an essential property in many biotechnological fields, such as enzyme design or protein-hybrid optoelectronics. While enzymes operating at high temperatures can potentially accelerate chemical reactions (1–3), the goal of protein-hybrid optoelectronics is to replace nonsustainable components with biogenic ones without losing device performance at operating temperatures above 70°C (4–7). It is therefore essential to accurately identify potentially thermostable proteins from naturally occurring or artificially engineered samples. To reduce the enormous search space and to guide researchers to potentially promising candidates, machine learning (ML) methods can be used to predict whether a protein is thermophilic or mesophilic.

Zhang and Fang (8) published one of the first ML studies to discriminate between thermophilic and mesophilic proteins, with a feedforward neural network performing best. Here, thermophilic proteins refer to proteins from organisms with an optimal growth temperature (OGT) >50°C, and mesophilic proteins come from organisms with an OGT between 20 and 40°C. A year later, they published one of the first benchmark datasets for thermophilicity prediction (9), for which they reported a validation accuracy of 87.4% for a support vector machine (SVM) with a radial basis function kernel and 86.6% for a boosting-based LogitBoost classifier. Gromiha and Suresh (10) reduced redundancy within the data of (9) by applying CD-HIT (11) with a threshold of 40% global sequence identity. They achieved a comparable performance for several ML-based methods, with slight advantages for an SVM, logistic regression and a neural network. Their results are consistent with the work of Lin and Chen (12). On a newly collected dataset, Lin and Chen showed a validation accuracy of 93.3% for ThermoPred, a predictor based on an SVM with a radial basis function kernel, slightly outperforming Random Forest and logistic regression. Several subsequent research papers also present SVMs with linear and nonlinear kernels as the best-performing models, often enhanced with additional feature engineering and selection (13–19). In a more recent publication, Charoenkwan *et al.* (20) introduced SCMTPP, a thermophilicity prediction model based on a scoring card method. On a unified dataset based on published data from (9,12,15), SCMTPP achieved a validation accuracy of 88.3% and outperformed ThermoPred by 0.5% on a test set containing 20% of the data with an accuracy of 86.5%. For iThermo, Ahmed *et al.* (21) used an analysis of variance-based feature selection and a three-layer multilayer perceptron (MLP) as a predictor. Similarly to SCMTPP, they outperformed ThermoPred on a newly collected dataset. SAPPHIRE (22) is a stacked ensemble learner based on six classification models, i.e. XGBoost, SVM with a linear and a radial basis function kernel, logistic regression, Random Forest and a partial least squares regression (PLS)-based classifier. Each prediction model was trained on 12 different feature sets using the same unified dataset as for SCMTPP. Furthermore, a genetic algorithm was trained to select 12 of the 72 predictions for a PLS-based meta-predictor. With an accuracy of 94.2%, SAPPHIRE outperforms SCMTPP (86.5%) on the test data used in (20). Recently, DeepTP, a deep learning-based model that uses both the amino acid sequence directly and physicochemical features, has been published. The sequence information is processed by a convolutional network to extract local features and a bidirectional long short-term memory (LSTM) network to account for long-range dependencies. After a self-attention layer, the output is concatenated with the normalized physicochemical features to serve as input for an MLP classification head. The authors report that DeepTP outperforms SAPPHIRE, SCMTPP and iThermo on a newly collected test set (23). BertThermo retrieves features directly from protein sequences using a large pretrained language model, namely ProtBert-BFD (24). Then, they further process the sequences using the synthetic minority oversampling technique to address data imbalance, and select features with a light gradient boosting machine. Finally, they apply logistic regression to generate predictions (25).

Although several studies suggest good predictive performance in discriminating thermophilic from nonthermophilic proteins, most of these studies report evaluation metrics only on validation data in a cross-validation setup or on randomly selected rather than species-specific test data. The importance of data collection and careful stratification of protein data for the generalization abilities of predictive models has been reviewed in detail in (26). In this study, the authors discussed two types of circularity that can lead to overly optimistic prediction results and reduced generalization ability of tools designed to predict the pathogenicity of missense variants. While the first type of circularity describes potential biases due to overlapping training and test data, the second type describes potential biases due to different variants of the same protein occurring in the training and in the evaluation of pathogenicity prediction tools. Similar biases can occur in thermophilicity prediction when different proteins from the same species occur in both the training and test data. For this reason, not only nested cross-validation metrics should be reported, but also evaluations on an independent test set containing only species that do not overlap with the species in the training data.

With respect to encoding protein sequences, i.e. representing the information they contain as numerical values such that subsequent prediction models can process them, various options exist, without yielding a superior approach so far (27,28). Most published thermophilicity prediction methods rely on feature engineering, e.g. by determining physicochemical properties. They do not directly use the protein sequence itself, e.g. via language models, which can lead to a loss of information. Recently, prediction methods using protein language models have been shown to have superior performance in several downstream tasks, such as the prediction of secondary structures, signal peptides or the binding of proteins to ligands (24,29–38).

In this work, we present a novel **Pro**tein **La**nguage model-based **Therm**ophilicity predictor (ProLaTherm). To evaluate the usefulness of protein language model embeddings for this prediction task, we benchmark ProLaTherm against several feature- and sequence-based comparison partners, including modern transformer-based architectures, as well as several prediction models from the literature. In contrast to the existing literature, we evaluate the performance not only in a nested cross-validation setup but also using test data containing only proteins from species that were not present during training. In this way, we ensure that the generalization ability of the models is estimated as unbiased as possible. In addition, we publish a new benchmark dataset for thermophilicity prediction tasks, which is based on a significant update of existing and newly collected data.

## Materials and methods

In the following, we first describe the data collection. Next, we outline the architecture of ProLaTherm, its comparison partners and the hyperparameter optimization. Finally, we summarize the preprocessing steps and experimental settings of this study.

### Data

In this study, we consider three commonly used benchmark datasets for protein thermophilicity prediction as well as newly collected data. Zhang and Fang (9) provided a dataset with 9422 UniProt identifiers and 9363 corresponding amino acid sequences from 16 thermophilic and 16 mesophilic organisms. To avoid potentially outdated identifiers and sequences, we checked the provided data against a recent UniProt release (2022_04, published October 2022) (39). Using the identifiers provided, we obtained data for 7684 of the 9422 proteins. For the remaining ones, for which Zhang and Fang (9) provided amino acid sequences, we used the NCBI protein BLAST (40) to obtain the corresponding current identifiers. We further checked the BLAST selected identifiers against UniProt’s historical data to ensure that they matched the original proteins. Finally, we obtained 9412 proteins with their corresponding UniProt identifiers, of which 3724 and 5688 are thermophilic and mesophilic, respectively.

Another commonly used dataset for thermophilicity prediction was constructed by Lin and Chen (12). To ensure that nonthermophilic proteins denature in the temperature ranges of thermophilic proteins, the authors chose 60°C as the lower limit for thermophilic organisms and 30°C as the upper limit for nonthermophilic ones. Their publicly available data contain 915 thermophilic and 793 nonthermophilic proteins, but with custom identifiers. To retrieve the corresponding UniProt identifiers for all provided sequences, we used NCBI’s protein BLAST and updated the data accordingly.

We also included data collected by Ahmed *et al.* (21), consisting of 1368 thermophilic and 1443 nonthermophilic proteins based on the thresholds of (12). Again, we checked all provided protein identifiers and sequences by downloading the latest versions from UniProt. For 55 proteins, for which we did not find a UniProt entry using the provided identifier, we used the NCBI protein BLAST and UniProt historical data as described above.

In addition, we collected a new dataset containing thermophilic and nonthermophilic proteins for a more diverse set of species. For this purpose, we selected suitable prokaryotic species from the TEMPURA database of growth temperatures (41) and extracted the corresponding proteins from UniProt. Following (12), we selected an OGT of 60°C as the lower limit and 30°C as the upper limit for thermophilic and nonthermophilic species, respectively.

For this study, we merged all three updated datasets from the literature with our newly collected data. This results in providing an enhanced and up-to-date benchmark dataset, which ensures updateability by including UniProt identifiers in contrast to existing ones (42). Furthermore, the inclusion of new data from a diverse set of species allows us to assess the generalization ability of the prediction models on unseen species. In the following, we refer to the data consisting of mesophilic proteins from (9) and nonthermophilic proteins from (12,21), and our newly collected data as nonthermophilic. Similarly to (12,21), we removed all proteins that were predicted or inferred by homology and kept only sequences consisting of the 20 proteinogenic amino acids. Excluding duplicates based on the UniProt identifier, this resulted in a final dataset containing a total of 2864 thermophilic and 4545 nonthermophilic proteins, collected from 91 and 173 species, respectively. From these data, 947 thermophilic proteins (belonging to 56 species) and 2245 nonthermophilic proteins (belonging to 85 species) come from our newly collected data. A list of all species with the number of protein sequences can be found in Supplementary Tables S2–S4. All data including meta-information are publicly available at https://github.com/grimmlab/ProLaTherm.

### Prediction models

In the following, we first describe the protein language model-based thermophilicity predictor ProLaTherm. With respect to the subsequent comparison partners, we distinguish based on the processing of the input protein sequences. First, we consider feature-based models that rely on manually engineered features, such as physicochemical properties. Second, we include hybrid sequence-based models that use amino acid features to learn sequence embeddings. Third, we consider approaches that are purely sequence-based, similarly to ProLaTherm, but in contrast train sequence embeddings from scratch. Finally, we outline the hyperparameter optimization.

#### ProLaTherm

Let $\boldsymbol{\rho }$ be a protein sequence consisting of *l*_*ρ*_ amino acids $a_1, \dots , a_{l_\rho }$ with *a*_*j*_ ∈ {A, C, D, E, F, G, H, I, K, L, M, N, P, Q, R, S, T, V, W, Y}. Denote by *y* ∈ {0, 1} the label of the protein sequence $\boldsymbol{\rho }$, with 1 indicating a thermophilic protein. In a first step, all amino acids *a*_*j*_ are ordinal encoded to $a_j^{\prime }\in \lbrace 1,\ldots ,20\rbrace$ and a sequence embedding $\boldsymbol{\Phi }\in \mathbb {R}^{l_{\rho }\times d}$ with *d* = 1024 is generated using a lookup embedding $\lbrace 1,\ldots ,20\rbrace \rightarrow \mathbb {R}^{d}$. The sequence embedding $\boldsymbol{\Phi }$ is further processed by the encoder of the ProtT5XLUniRef50 model to retrieve the protein language model embedding $\boldsymbol{\Phi }^{\ast } \in \mathbb {R}^{l_{\rho }\times d}$:


\begin{eqnarray*} \boldsymbol{\Phi }^{\ast } = \texttt {ProtT5XLUniRef50\_Encoder}(\boldsymbol{\Phi }). \end{eqnarray*}

For the feature extractor ProtT5XLUniRef50, the encoder part of a T5-3B model (43), a transformer-based model consisting of 24 layers with a 32-head self-attention, is integrated. ProtT5XLUniRef50 was pretrained for protein sequence reconstruction in a self-supervised setup by Elnaggar *et al.* (24) on UniRef50 (44). We utilize latent representations from this pretrained protein language model, as it has been shown that these are able to capture meaningful biophysical features (24). Thus, these cross-domain embeddings could improve the generalization abilities of a protein thermophilicity classifier. To obtain an input vector for the feedforward head classifier, we apply an average pooling on the protein language model embedding $\boldsymbol{\Phi }^{\ast }$ over the sequence length *l*_*ρ*_, resulting in a *d*-dimensional vector $\boldsymbol{\mu }^{\ast } \in \mathbb {R}^d$. With the transposed *k*th row vector of $\boldsymbol{\Phi }^{\ast }$ denoted as $(\boldsymbol{\phi }_k^{\ast })^{\rm T}$, the average pooling along *l*_*ρ*_ is defined as


\begin{eqnarray*} \boldsymbol{\mu }^{\ast } = \frac{1}{l_{\rho }}\sum _{k=1}^{l_{\rho }} (\boldsymbol{\phi }_k^{\ast })^{\rm T}. \end{eqnarray*}

**Figure 1.**
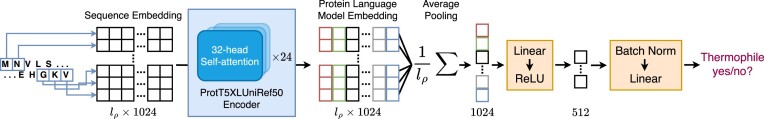
Overview of ProLaTherm. After deriving sequence embeddings from the protein sequence, we retrieve protein language model embeddings by using the encoder part of ProtT5XLUniRef50. These are further average pooled along the sequence length to be processed by the head classifier. The head classifier consists of a fully connected layer with rectified linear activation, followed by batch normalization and a fully connected output layer. We further apply dropout before the average pooling and output layer.

The head classifier starts with a fully connected layer with a rectified linear unit (ReLU) as nonlinear activation function and reduces the dimensionality *d* of $\boldsymbol{\mu }^{\ast }$ to *d*/2 = 512. This is followed by batch normalization (45) and a fully connected output layer yielding logits of the final predictions. An overview of ProLaTherm is summarized in Figure 1.

The head classifier was trained in a supervised fashion minimizing the cross-entropy loss $\mathcal {L}$ between the label *y*_*i*_ of the *i*th protein in a batch of size *n*_b_ and a predicted probability for thermophilicity *p*_*i*_ given as


\begin{eqnarray*} \mathcal {L} = - \frac{1}{n_{\rm b}} \sum _{i=1}^{n_{\rm b}} y_i \log (p_i) + (1 - y_i) \log (1-p_i). \end{eqnarray*}

The Adam optimizer was used for model training while treating the learning rate as a hyperparameter and reducing it on a plateau of the validation loss (46). For regularization, we apply dropout before the average pooling and the output layer (47) and use early stopping; i.e. the optimization process is terminated if the loss on a validation set does not improve for a certain period. Further details on the hyperparameter optimization are outlined later in this section. An overview of all hyperparameters and ranges is shown in Supplementary Table S5.

#### Feature-based comparison partners

To benchmark ProLaTherm, we include five feature-based classifiers, ranging from classical ML models over ensemble learners to a neural network-based architecture. These models rely on manual feature engineering, which summarizes information from the protein sequences, such as physicochemical properties. These features are described in more detail in the ‘Experimental settings’ section, Supplementary Methods and Supplementary Table S1. An overview of all optimized hyperparameters and ranges can be found in Supplementary Table S6.

As a first comparison partner, we use Elastic Net, a logistic regression model with a penalty term using a weighted sum of the L1 and the L2 norms (48). This regularization combines the automatic feature selection effect of the L1 norm with the weight distribution among correlated features due to the L2 norm. The strength of the regularization term and the ratio between the L1 and the L2 norms are optimized during the hyperparameter search. As summarized in the ‘Introduction’ section, SVMs (49) have shown good performance in several thermophilicity prediction studies. Therefore, we also include an SVM in our study. An essential component of an SVM is the kernel function, for which we consider a linear and a radial basis function as well as a polynomial kernel. Besides the choice of the kernel function, the strength of the regularization term, which penalizes complex models, and kernel-related parameters are tuned in the hyperparameter search.

We also include the two ensemble learners: Random Forest and XGBoost. Random Forest uses bagging, i.e. aggregating the predictions of weak learners trained on random subsamples of the training data (50). In contrast, XGBoost uses gradient boosting, which greedily adds prediction models focusing on misclassifications of the current ensemble (51).

In addition, we implement an MLP, for which the number of layers and neurons in each layer is optimized during the hyperparameter search. We add batch normalization and dropout after each fully connected layer and a fully connected output layer. For the nonlinear activation function, we choose between a ReLU and a hyperbolic tangent. Similarly to ProLaTherm, we minimize the cross-entropy loss and employ the Adam optimizer with an optimized learning rate and a learning rate scheduling (46).

#### Hybrid sequence-based comparison partners

LSTM_BasicDesc and Bi-LSTM_BasicDesc can be thought of as hybrid sequence-based models that use features of the amino acids for the input embeddings. We encode each amino acid with normalized values of physicochemical properties, i.e. weight, charge, polarity, aromaticity, hydrophobicity and van der Waals volume. Our goal is to integrate physicochemical properties as additional input information, which we neglect in the case of purely sequence-based embeddings learned from scratch. The six basic descriptors lead to a low-dimensional embedding compared to the other sequence-based approaches. To potentially enhance the input of the LSTM network part, we therefore increase the dimensionality of the latent representation using fully connected layers with nonlinear activation and dropout. These hybrid latent representations are further processed by an LSTM and Bi-LSTM, respectively. Both LSTM_BasicDesc and Bi-LSTM_BasicDesc are designed with an optimized number of LSTM layers including a dropout mechanism, with the Bi-LSTM employing a bidirectional architecture. Then, the last hidden state from the LSTM network is extracted and further processed by the same head classifier architecture as for ProLaTherm. Both methods are trained using the Adam optimizer minimizing the cross-entropy loss, with the learning rate considered as a hyperparameter and a learning rate scheduling based on the validation loss. Early stopping is applied to avoid overfitting.

#### Purely sequence-based comparison partners

Furthermore, we consider five comparison partners that, similarly to ProLaTherm, directly use the protein sequence as input. In contrast to ProLaTherm, we do not use a pretrained feature extractor, but train the sequence embeddings from scratch, without physicochemical information. Hyperparameters and ranges for all sequence-based comparison partners are given in Supplementary Tables S7 and S8. For MLP_Embedding, LSTM and Bi-LSTM, we use an ordinal encoding followed by an embedding layer to transform the amino acid sequence into a sequence of real-valued vectors (52,53). Regarding MLP_Embedding, we apply the same architecture as for the head classifier of ProLaTherm, but choose whether to include the first fully connected layer during hyperparameter optimization. The LSTM and Bi-LSTM employ the same architecture as LSTM_BasicDesc and Bi-LSTM_BasicDesc, respectively. An optimized number of LSTM layers including a dropout mechanism, with the Bi-LSTM employing a bidirectional architecture, is followed by the head classifier architecture of ProLaTherm using the last hidden state of the recurrent network part.

With vanilla-Transformer and BigBird, we add two transformer-based architectures, similarly to ProtT5XLUniRef50 (54,55). For both, we again use an ordinal encoding and embedding layer to transform the amino acid sequence. Multi-head self-attention, a key element of a transformer-based architecture, is permutation equivariant. Hence, we preserve the positional information with an additional positional embedding layer. The sum of the outputs of both embedding layers serves as the input of vanilla-Transformer and BigBird. For computational efficiency in terms of runtime and memory, we employ an average pooling layer reducing the sequence length prior to the transformer layers in vanilla-Transformer. As BigBird uses a sparse attention mechanism scaling linearly with the sequence length instead of quadratically, the average pooling is not needed. Hence, comparing the results of vanilla-Transformer and BigBird can be used to evaluate whether using the full sequence length as input of the transformer-based layers is beneficial. vanilla-Transformer and BigBird consist of an optimized number of transformer layers, each comprising multiple self-attention heads, with the number of heads considered as a hyperparameter (see Supplementary Table S8). The transformer layers of both prediction models follow a pre-layer normalization design, with layer normalization followed by a multi-head self-attention and a feedforward layer, as well as skip connections around the self-attention and feedforward parts (56). However, vanilla-Transformer and BigBird differ regarding the self-attention mechanism. For vanilla-Transformer, we employ the regular self-attention calculation with each sequence element attending to all others. BigBird instead applies an approximation, where each sequence element only attends to a subset of the other sequence elements, realized via local and global tokens. Similarly to ProLaTherm, we apply an average pooling along the sequence length on the output of the last transformer layer in both cases. Finally, we use the head classifier design of ProLaTherm.

All sequence-based models are trained from scratch to minimize the cross-entropy loss using the Adam optimizer with an optimized learning rate and a learning rate reduction on a validation loss plateau. In addition to dropout, we apply early stopping for regularization.

#### Comparison partners from the literature

For a comparison with state-of-the-art protein thermophilicity prediction models from the literature, we include six publicly available predictors, i.e. ThermoPred (12), SCMTPP (20), iThermo (21), SAPPHIRE (22), DeepTP (23) and BertThermo (25), which we summarized in the ‘Introduction’ section.

#### Hyperparameter optimization

For hyperparameter optimization, we apply state-of-the-art Bayesian optimization (57) using the Python package Optuna (58). Bayesian optimization attempts to guide the search toward more promising parameter candidates based on the performance of already tested parameter sets. Defining an objective value based on validation data enables the formulation of a probabilistic model mapping from parameter candidates to a probability of an objective value. This probabilistic model then allows the selection of promising parameter settings for subsequent trials. In comparison to grid or random search (59), Bayesian optimization is computationally more expensive in terms of suggesting hyperparameter values but potentially converges earlier as the parameter choice might be superior (60). We perform 200 optimization trials for each model. Furthermore, we stop nonpromising trials if intermediate results, i.e. the performance on cross-validation folds, are worse than the 80th percentile of previous runs at the same step.

### Experimental settings

#### Data preparation

In our study, we consider two different experimental setups: (i) to assess the generalization ability of the used models based on an unbiased empirical performance estimate, we use a nested cross-validation on the complete dataset; and (ii) to evaluate whether the used models tend to only predict the species or are actually able to predict thermophilicity, we conduct a cross-validation with an additional species-specific test set. For this test set, we only use protein sequences from species that are not present during the training. The results on this test set then allow to evaluate whether a prediction model generalizes across species. For both experimental setups, we use our new benchmark dataset described above.

For the nested cross-validation, we first removed all proteins shorter than the 5th percentile or longer than the 95th percentile from the benchmark dataset. In accordance with (10,12), we then used CD-HIT (11) with a threshold of 40% global sequence identity to filter out highly similar sequences. This preprocessing step led to a dataset consisting of 1699 thermophilic and 3440 nonthermophilic proteins. We then split this dataset into three outer folds and five inner folds. Due to the class imbalance, we applied class-stratified splits to ensure a similar class distribution.

To construct a test set containing only proteins from species not present during training, we selected data of species that only occur in our newly generated dataset. By doing so, we also ensure that there is no overlap between this species-specific test set and the benchmark datasets from the literature, which were used to train the comparison partners from the literature. We again filtered based on the sequence length as described above and used CD-HIT with a 40% cutoff on the sequences of the remaining species. Hence, we obtained 1539 thermophilic and 3258 nonthermophilic proteins for training and validation, which we split in a class-stratified 5-fold cross-validation. The independent test set, subsequently called *test set 1*, contains 345 and 224 proteins from 51 thermophilic and 75 nonthermophilic species, respectively. Beyond that, we further analyze whether the generalization of the prediction models across species is influenced by the evolutionary relationship between the test and training data. For this purpose, we used BLAST to identify protein sequences in *test set 1* that are evolutionary less related to the training data. We only considered proteins with an identity <50%, resulting in *test set 2* containing 203 thermophilic and 192 nonthermophilic out of 345 and 224 protein sequences, respectively. A summary containing the number of proteins in each dataset is shown in Table 1. Besides the raw data, we provide files containing all data splits as well as meta-information with respect to each sample in our GitHub repository: https://github.com/grimmlab/ProLaTherm/tree/main/data.

**Table 1.** Number of thermophilic and nonthermophilic proteins per dataset

Dataset	# Thermo.	# Nonthermo.
Total collected data	2864	4545
Nested cross-validation data	1699	3440
Species-specific cross-validation data	1539	3258
Test set 1	345	224
Test set 2	203	192

The rows show the number of proteins in the entire dataset and the data used in our two experimental setups after the respective preprocessing steps. The nested cross-validation data refer to all proteins used for training and testing in our nested cross-validation experiment. The species-specific cross-validation data, test set 1 and test set 2 are part of the species-specific experiment. The former was used for training and validation, whereas the latter two are the two sets with distinct species not present in the species-specific cross-validation data.

The training data of the comparison partners from the literature overlap partly with the published datasets that we updated for our new benchmark dataset, which might not allow an unbiased comparison with our nested cross-validation experiment. To ensure a fair comparison, we evaluate these comparison partners using the independent test data (*test set 1* and *test set 2*), which consist of newly collected protein sequences. For DeepTP, we excluded the results on 25 protein sequences from evaluation metric calculation due to an overlap with their training data, whereas there is no overlap for the other comparison partners.

#### Feature engineering for feature-based competitors

For the feature-based comparison partners, we derived a total of 599 features, describing physicochemical and structural properties of the protein sequences. To compute those features, we used the Python package iFeatureOmega (61,62). An overview of all included descriptors can be found in Table 2, while we refer to Supplementary Section S1 for detailed explanations of the different features.

**Table 2.** Overview of all included features

Descriptor group	Descriptor	# Features
Basic descriptors	Weight	1
	Charge	3
	Polarity	2
	Aromaticity	1
	Mean hydrophobicity	1
	Mean van der Waals volume	1
Residue composition	Amino acid composition	20
	Dipeptide composition	400
Physicochemical	Composition	21
properties	Transition	21
	Distribution	105
Sequence order effects	Pseudo amino acid composition	23

Descriptors are clustered in four groups given in the first column. The number of features for each descriptor is shown in the last column, summing up to 599 features in total. Detailed explanations for all descriptors can be found in Supplementary Data.

#### Evaluation

For evaluation, we consider the commonly used metrics accuracy, precision, recall, specificity, balanced accuracy (BACC), *F*1-score and Matthew’s correlation coefficient (MCC) (63). For *n*_s_ samples, tp true positives (both the label and the prediction are thermophilic), tn true negatives (both the label and the prediction are nonthermophilic), fp false positives (the label is nonthermophilic but the prediction is thermophilic) and fn false negatives (the label is thermophilic but the prediction is nonthermophilic), these metrics are defined as follows:


\begin{eqnarray*} \begin{aligned} {\rm accuracy} &= \frac{{\rm tp} + {\rm tn}}{n_{\rm s}}, \\ {\rm precision} &= \frac{{\rm tp}}{{\rm tp} + {\rm fp}}, \\ {\rm recall} &= \frac{{\rm tp}}{{\rm tp} + {\rm fn}}, \\ {\rm specificity} &= \frac{{\rm tn}}{{\rm tn} + {\rm fp}}, \\ {\rm BACC} &= \frac{1}{2} ({\rm recall} + {\rm specificity}),\\ F1{\hbox{-}}{\rm score} &= \frac{2 \times {\rm precision} \times {\rm recall}}{{\rm precision} + {\rm recall}}, \\ {\rm MCC} &= \frac{{\rm tp} \times {\rm tn} - {\rm fp} \times {\rm fn}}{\sqrt{({\rm tp}+{\rm fp})({\rm tp}+{\rm fn})({\rm tn}+{\rm fp})({\rm tn}+{\rm fn})}}. \\ \end{aligned} \end{eqnarray*}

Since the MCC is a robust evaluation metric, e.g. in case of class imbalance, we take the MCC as our main criterion. In contrast to the other four metrics that lie in the range [0, 1], the MCC returns a value between −1 and 1 with 1 reflecting perfect predictions, 0 uniformly random predictions and −1 a totally disagreeing predictor. Precision gives the ratio of the actual thermophilic proteins among the instances that were predicted to be thermophilic, whereas recall shows the ratio of the thermophilic proteins that the prediction model correctly recognized. From a practical perspective, both measures are important. A low precision could lead to unnecessarily spending lab resources on nonthermophilic proteins, and a low recall would result in discarding actual thermophilic proteins. We further consider receiver operating characteristic (ROC) curves, which plot the true positive rate against the false positive rate.

For both the nested cross-validation and the experiment with the independent test sets containing nonoverlapping species, we use the MCC averaged across the five inner folds as the objective value of the Bayesian optimization and the MCC on each fold as intermediate results for a potential pruning. At the end of each 5-fold inner cross-validation, we select the best hyperparameter setting and retrain the prediction model on the training and validation data. Finally, we test each prediction model on independent data—either on one of the outer folds in case of the nested cross-validation or on the test set containing nonoverlapping species. All optimizations were performed in Python 3.8. The source code and data are available at https://github.com/grimmlab/ProLaTherm.

## Results and discussion

### Result summary

As described in the ‘Experimental settings’ section, we consider two different experimental setups: (i) a 3-fold nested cross-validation with five inner folds on the whole dataset and (ii) a 5-fold cross-validation with a hold-out test set containing only species that do not occur within the training data. Regarding the prediction models, we can distinguish between three different settings: (i) feature-based models relying on manually engineered features; (ii) hybrid sequence-based models using amino acid properties to learn a hybrid sequence embedding; and (iii) purely sequence-based models, either learning sequence embeddings from scratch or, in case of ProLaTherm, leveraging embeddings from a pretrained protein language model. Detailed results of the whole hyperparameter optimizations for all models and both experiments can be found in our GitHub repository: https://github.com/grimmlab/ProLaTherm.

In Table 3, we summarize the results of the nested cross-validation experiment for different evaluation metrics. The protein language model-based classifier ProLaTherm shows superior performance with respect to all evaluation metrics. Regarding MCC, ProLaTherm outperforms all comparison partners with an improvement of at least 18.1%. Feature-based classifiers (except for Random Forest) perform similarly and tend to have slightly better predictive performance than sequence-based prediction models. Among the sequence-based comparison partners, transformer-based models (vanilla-Transformer and BigBird) and a hybrid model consisting of a Bi-LSTM using basic amino acid descriptors (Bi-LSTM_BasicDesc) perform best and almost as good as models relying on manual feature engineering. As both hybrid models (LSTM_BasicDesc and Bi-LSTM_BasicDesc) perform better than their purely sequence-based counterparts (LSTM and Bi-LSTM), integrating physicochemical properties to learn the embeddings seemed to be beneficial.

**Table 3.** Overview of the test results on the nested cross-validation using the full dataset

Prediction model	Accuracy	*F*1-score	Precision	Recall	Specificity	BACC	MCC
**Feature-based models**							
Elastic Net	0.900 ± 0.002	0.842 ± 0.006	0.881 ± 0.015	0.808 ± 0.022	0.946 ± 0.009	0.877 ± 0.007	0.771 ± 0.006
SVM	0.900 ± 0.001	0.843 ± 0.003	0.880 ± 0.012	0.809 ± 0.014	0.945 ± 0.007	0.877 ± 0.004	0.771 ± 0.002
Random Forest	0.867 ± 0.005	0.768 ± 0.010	0.904 ± 0.004	0.667 ± 0.013	0.965 ± 0.001	0.816 ± 0.007	0.692 ± 0.012
XGBoost	0.908 ± 0.005	0.855 ± 0.008	0.896 ± 0.013	0.818 ± 0.019	0.953 ± 0.007	0.885 ± 0.007	0.790 ± 0.011
MLP	0.902 ± 0.003	0.843 ± 0.007	0.896 ± 0.006	0.796 ± 0.018	0.954 ± 0.004	0.875 ± 0.007	0.775 ± 0.008
**Hybrid sequence-based models**							
LSTM_BasicDesc	0.877 ± 0.006	0.795 ± 0.022	0.891 ± 0.040	0.723 ± 0.064	0.954 ± 0.023	0.838 ± 0.021	0.719 ± 0.014
Bi-LSTM_BasicDesc	0.891 ± 0.001	0.829 ± 0.004	0.860 ± 0.009	0.802 ± 0.014	0.935 ± 0.006	0.868 ± 0.004	0.751 ± 0.004
**Purely sequence-based models**							
MLP_Embedding	0.871 ± 0.019	0.803 ± 0.013	0.824 ± 0.078	0.795 ± 0.056	0.908 ± 0.055	0.852 ± 0.006	0.713 ± 0.030
LSTM	0.866 ± 0.021	0.768 ± 0.064	0.890 ± 0.073	0.697 ± 0.132	0.949 ± 0.040	0.823 ± 0.048	0.697 ± 0.045
Bi-LSTM	0.871 ± 0.014	0.798 ± 0.024	0.842 ± 0.079	0.775 ± 0.094	0.919 ± 0.056	0.847 ± 0.023	0.713 ± 0.023
vanilla-Transformer	0.883 ± 0.009	0.802 ± 0.020	0.913 ± 0.020	0.716 ± 0.039	0.966 ± 0.009	0.841 ± 0.016	0.732 ± 0.021
BigBird	0.884 ± 0.004	0.811 ± 0.004	0.876 ± 0.023	0.756 ± 0.017	0.947 ± 0.013	0.851 ± 0.003	0.732 ± 0.008
ProLaTherm	**0.970 ± 0.004**	**0.955 ± 0.005**	**0.963 ± 0.015**	**0.947 ± 0.005**	**0.982 ± 0.008**	**0.964 ± 0.002**	**0.933 ± 0.008**

Each cell shows the mean and standard deviation across the three outer folds for the given evaluation metric and prediction model. The prediction models are grouped as feature-based as well as hybrid and purely sequence-based. The best result for each evaluation metric is highlighted in bold. ProLaTherm outperforms all comparison partners.

**Figure 2.**
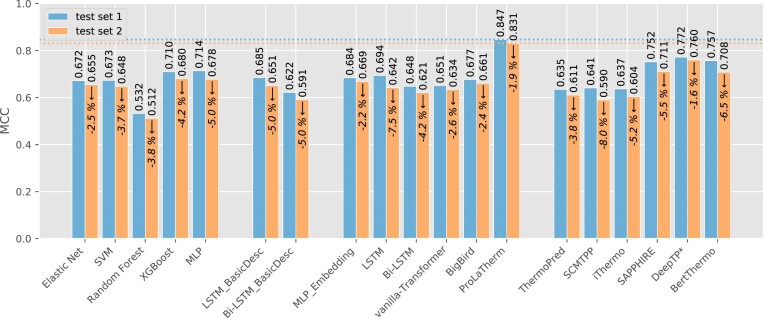
Overview of the test results with no overlap between the species in the test and cross-validation data: For each prediction model, we show the MCC on the full test set (*test set 1*) as well as on the samples that are evolutionary less related to the training data (*test set 2*). The prediction models are grouped as feature-based as well as hybrid and purely sequence-based. We further show comparison partners from the literature. The best result for both test sets is marked by a horizontal dotted line. Furthermore, the percentage change in MCC on *test set 2* is given for each prediction model. For DeepTP, 25 proteins were excluded from the evaluation metric calculation due to overlap with the comparison partner’s training data. For a full overview of the results with respect to all evaluation metrics, we refer to Table 4, and to Supplementary Table S9 for a comparison of validation and test results.

**Figure 3.**
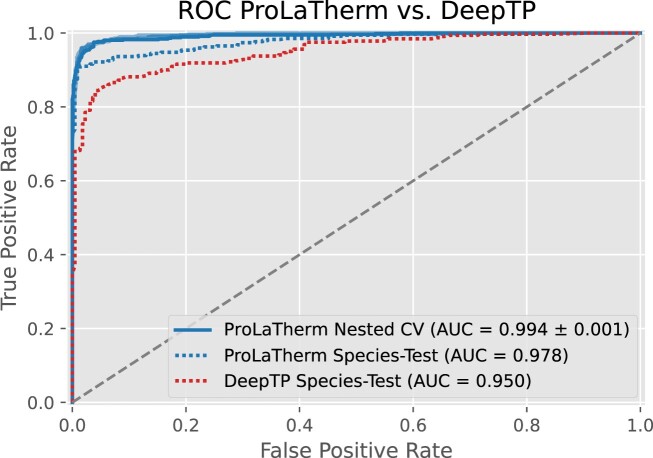
ROC curves of ProLaTherm and DeepTP. For ProLaTherm, we show the two ROC curves, one for the nested cross-validation experiment and one for the independent test set containing only species that do not occur in the training data (*test set 1*). Further, we show the ROC curve of DeepTP, the second-best performing prediction model in terms of MCC.

In the second experiment, we evaluate the generalization abilities on an independent test set consisting of distinct species not present during training (*test set 1* and *test set 2*). On these data, we also include state-of-the-art thermophilicity prediction models from the literature, i.e. ThermoPred (12), SCMTPP (20), iThermo (21), SAPPHIRE (22), DeepTP (23) and BertThermo (25), since we can ensure a fair comparison as outlined in the ‘Experimental settings’ section. Results for all prediction models and evaluation metrics, both on test set 1 and on *test set 2*, can be found in Table 4. We further summarize both test results in terms of MCC in Figure 2 for easier readability. For a comparison of cross-validation results and the performance on *test set 1*, we refer to Supplementary Table S9. Regarding *test set 1*, we observe that ProLaTherm again outperforms all comparison partners, except for DeepTP on recall. DeepTP shows a tendency toward predicting a protein to be thermophilic, as indicated by a high recall and a comparably low precision. In contrast to the nested cross-validation, we do not see clear advantages for the feature-based over the sequence-based classifiers, even though MLP and XGBoost are ranked after ProLaTherm, DeepTP and SAPPHIRE for MCC. Furthermore, the two hybrid models are outperformed by LSTM and Bi-LSTM in terms of MCC. For all prediction models, we observe a drop in predictive performance (except for precision) between validation data and the test set containing only species seen for the first time (see Supplementary Table S9). This is not surprising, since generalizing predictions to proteins from unknown species is a seemingly more difficult task. With respect to MCC, the drop in performance is between 9.3% for MLP_Embedding and 24.1% for Random Forest. Also for ProLaTherm, we observe a drop of 10.8%. Nevertheless, ProLaTherm still outperforms all other models, including classifiers from the literature, by at least 9.7% (DeepTP). The ROC curves for ProLaTherm and DeepTP, the second-best competitor on the unseen species, confirm a superior performance of ProLaTherm, as shown in Figure 3. The remaining prediction models from the literature, except for SAPPHIRE, DeepTP and BertThermo, perform worse than several of the feature- and sequence-based models in terms of MCC.

**Table 4.** Overview of the test results with no overlap between the species in the test and cross-validation data

Prediction model	Accuracy	*F*1-score	Precision	Recall	Specificity	BACC	MCC
**Feature-based models**							
Elastic Net	0.814 [0.808]	0.822 [0.776]	0.976 [0.964]	0.710 [0.650]	0.973 [0.974]	0.842 [0.812]	0.672 [0.655]
SVM	0.817 [0.808]	0.827 [0.780]	0.969 [0.944]	0.722 [0.665]	0.964 [0.958]	0.843 [0.812]	0.673 [0.648]
Random Forest	0.712 [0.716]	0.695 [0.632]	0.969 [0.950]	0.542 [0.473]	0.973 [0.974]	0.758 [0.723]	0.532 [0.512]
XGBoost	0.840 [0.823]	0.852 [0.798]	0.974 [0.965]	0.757 [0.680]	0.969 [0.974]	0.863 [0.827]	0.710 [0.680]
MLP	0.844 [0.823]	0.856 [0.799]	0.971 [0.959]	0.765 [0.685]	0.964 [0.969]	0.865 [0.827]	0.714 [0.678]
**Hybrid sequence-based models**							
LSTM_BasicDesc	0.837 [0.818]	0.854 [0.803]	0.934 [0.902]	0.786 [0.724]	0.915 [0.917]	0.850 [0.820]	0.685 [0.651]
Bi-LSTM_BasicDesc	0.779 [0.767]	0.781 [0.716]	0.974 [0.959]	0.652 [0.571]	0.973 [0.974]	0.813 [0.773]	0.622 [0.591]
**Purely sequence-based models**							
MLP_Embedding	0.819 [0.813]	0.827 [0.781]	0.984 [0.978]	0.713 [0.650]	0.982 [0.984]	0.848 [0.817]	0.684 [0.669]
LSTM	0.837 [0.810]	0.851 [0.790]	0.953 [0.916]	0.768 [0.695]	0.942 [0.932]	0.855 [0.813]	0.694 [0.642]
Bi-LSTM	0.807 [0.795]	0.818 [0.767]	0.950 [0.924]	0.719 [0.655]	0.942 [0.943]	0.830 [0.799]	0.648 [0.621]
vanilla-Transformer	0.803 [0.797]	0.812 [0.765]	0.964 [0.949]	0.701 [0.640]	0.960 [0.964]	0.831 [0.802]	0.651 [0.634]
BigBird	0.814 [0.808]	0.821 [0.774]	0.984 [0.977]	0.704 [0.640]	0.982 [0.984]	0.843 [0.812]	0.677 [0.661]
ProLaTherm	**0.919** [**0.909**]	**0.929** [**0.903**]	**0.997** [**0.994**]	0.870 [0.828]	**0.996** [**0.995**]	**0.933** [**0.911**]	**0.847** [**0.831**]
**Comparison partners from the literature**							
ThermoPred (12)	0.817 [0.803]	0.840 [0.796]	0.895 [0.849]	0.791 [0.749]	0.857 [0.859]	0.824 [0.804]	0.635 [0.611]
SCMTPP (20)	0.807 [0.782]	0.821 [0.756]	0.937 [0.893]	0.730 [0.655]	0.924 [0.917]	0.827 [0.786]	0.641 [0.590]
iThermo (21)	0.819 [0.800]	0.842 [0.796]	0.893 [0.837]	0.797 [0.759]	0.853 [0.844]	0.825 [0.801]	0.637 [0.604]
SAPPHIRE (22)	0.870 [0.846]	0.884 [0.831]	0.966 [0.949]	0.814 [0.739]	0.955 [0.958]	0.885 [0.849]	0.752 [0.711]
DeepTP^a^ (23)	0.888 [0.880]	0.903 [0.875]	0.925 [0.892]	**0.882** [**0.858**]	0.897 [0.901]	0.889 [0.879]	0.772 [0.760]
BertThermo (25)	0.880 [0.853]	0.898 [0.852]	0.931 [0.884]	0.867 [0.823]	0.902 [0.885]	0.884 [0.854]	0.757 [0.708]

For each prediction model, we show the given evaluation metric on full test set (*test set 1*) as well as on the samples that are evolutionary less related to the training data (*test set 2*) in square brackets. The prediction models are grouped as feature-based as well as hybrid and purely sequence-based. We further show comparison partners from the literature. The best result for each evaluation metric is highlighted in bold.

^a^ Twenty-five proteins excluded from evaluation metric calculation due to overlap with the comparison partner’s training data.

In order to further investigate the generalizability across all models, we reduced the test data by filtering out evolutionarily closer related proteins (*test set 2*; see the ‘Experimental settings’ section). Again, we detect a performance drop for almost all evaluation metrics and prediction models if we only consider proteins in our test set that are evolutionary less related to the training data (see Figure 2 and Table 4). Nevertheless, ProLaTherm still performs best with an MCC decreasing from 0.847 to 0.831 (relative drop of 1.9), whereas the second-best competitor DeepTP decreases from 0.772 to 0.760 (relative drop of 1.6%). Hence, ProLaTherm has a 9.3% better MCC than DeepTP, indicating a strong performance even for more challenging data.

### Prediction analyses of ProLaTherm

In the following, we further analyze the prediction results of the top-performing model ProLaTherm on *test set 1* containing species not included during training. In Table 5, we can observe that ProLaTherm yields 1 false positive and 45 false negatives, which also results in a higher precision than recall of 0.997 and 0.870, respectively. The single nonthermophilic protein predicted to be thermophilic by ProLaTherm is also misclassified by the best feature-based and sequence-based classifiers MLP and LSTM, respectively. The test data contain a total of 51 thermophilic species. We observe false negative predictions for 13 of these 51 species, but with 26 out of 45 false negative predictions, the majority of misclassifications occur for *Geobacillus kaustophilus*. This species, for which we have 37 protein sequences, also leads to the highest amount of false negatives for DeepTP (15), LSTM (24) and MLP (28). A potential reason for the high number of false negatives on *G. kaustophilus* can be found in Supplementary Table S10, in which we show the BLAST sequence identity of the thermophilic proteins with the best hit among the training data averaged across the species. Except for species with only one protein, *G. kaustophilus* has by far the highest mean identity with the nonthermophilic training data.

**Table 5.** Performance of ProLaTherm on the test set with nonoverlapping species for different sequence lengths

Seq. length	# Thermo.	# Nonthermo.	fp	fn	MCC
Total	345 (308)	224	1	45 (19)	0.847 (0.926)
[82, 208]	81 (72)	39	0	11 (5)	0.821 (0.908)
[209, 334]	110 (96)	67	1	19 (7)	0.789 (0.902)
[335, 460]	87 (81)	69	0	11 (5)	0.868 (0.935)
[461, 586]	43 (38)	38	0	4 (2)	0.906 (0.949)
[587, 712]	24 (21)	11	0	0 (0)	1.000 (1.000)

In each row, we show the number of thermophilic and nonthermophilic proteins, the number of false positives (fp) and false negatives (fn), and the MCC for the given range of the sequence length. In parentheses, we additionally show numbers without considering the species *G. kaustophilus*, for which the majority of false negatives occur.

In Table 5, we further assess a potential relationship between the amino acid sequence length and the prediction performance. For that purpose, we bin all proteins based on their sequence length into four equal-sized ranges and evaluate misclassifications, i.e. the number of false positives and false negatives, as well as the MCC. The MCC tends to increase for longer sequences, except for the range [209, 334] for which a single false positive leads to a decrease in the numerator of the MCC. If we do not consider *G. kaustophilus*, for which the majority of false negatives occur, the MCC still increases asymptotically with the sequence length. One reason for this improving performance might be that longer sequences provide more information for the protein language model to capture.

We further evaluate a potential relationship between misclassifications and the OGT, which we used to determine thermophilic species (minimum OGT of 60°C), shown in Table 6. We observe that nearly half of the proteins in the first OGT range from 60 to 70°C were wrongly classified as nonthermophilic. Compared with the other three ranges with a maximum ratio of false negatives of 8.3%, this is by far the highest value. For species with an OGT over 70°C, the false negative rate reduces from 13.0% to 2.6%. The OGT of *G. kaustophilus* is at the lower threshold to be labeled thermophilic. Nevertheless, even without considering this species, the misclassification rate in the range [60, 70) would still be 29.3%.

**Table 6.** Performance of ProLaTherm on the thermophilic species of the test set with nonoverlapping species for different OGTs

OGT (°C)	# Species	# Proteins	tp	fn
[60, 70)	15	78	40	38
[70, 80)	11	48	44	4
[80, 90)	19	181	179	2
90+	6	38	37	1

In each row, we show the number of species and proteins as well as the number of true positives and false negatives for the given range of the OGT.

### Discussion

In summary, the protein language model-based thermophilicity predictor ProLaTherm shows superior performance, both in a nested cross-validation setup and on an independent test set including only species not present in the training data. ProLaTherm outperforms the second-best competitor XGBoost by 18.1% regarding the mean test MCC on the nested cross-validation. The current state-of-the-art method DeepTP (23) is outperformed by ProLaTherm with 9.7% using the species-specific test set (*test set 1*). However, in general we observe a drop in predictive performance across all models when applied on the species-specific test set. When considering evolutionarily distantly related protein sequences (*test set 2*), ProLaTherm only shows a minor drop in performance compared to others. Interestingly, on *test set 1*, ProLaTherm yields only 1 false positive with a false positive rate of 0.4% but 45 false negatives resulting in a false negative rate of 13.0%. Thirty-eight of the 45 false negatives stem from species with an OGT between 60 and 70°C. Hence, ProLaTherm performs better on thermophilic proteins with a higher OGT over 70°C, with a false negative rate of 2.6%. More importantly, most of the false negative predictions are caused by 26 proteins from the species *G. kaustophilus*. The reason for this could be that the thermophilic species *G. kaustophilus* with an OGT of 62.5°C shows the highest mean identity with nonthermophilic protein sequences from the training data, when excluding species with only one protein sequence. DeepTP achieves the second-best performance on the independent test set. This confirms the authors’ study reporting a better performance compared with other protein thermophilicity prediction methods (23). In accordance with existing literature that shows superior performance on various protein-related prediction tasks for models leveraging protein language models (24,29–38), ProLaTherm performs best. In recent scientific publications, methods using embeddings from protein language models show a competitive performance for secondary and tertiary structure prediction without requiring multiple sequence alignment information while being computationally less demanding in the inference stage. Without access to labeled data during pretraining, the employed ProtT5XLUniRef50 was able to capture long-range inter-residue distances (24,64). Beyond that, it is known that amino acid interactions present in secondary and tertiary structures influence protein thermostability (65,66). Thus, it seems reasonable that ProLaTherm shows a superior performance.

The overall good predictive performance of ProLaTherm has important implications for many biotechnological domains. Because of ProLaTherm’s high precision, laboratory scientists can allocate their scarce resources more efficiently to the most promising candidates. Beyond that, ProLaTherm also achieves the second-highest recall among all tools, with a further improved prediction performance for proteins with higher OGTs. This high recall ensures that actually thermophilic candidates are not mistakenly excluded for further evaluation. To support biotechnological research, it is highly interesting to extend our work with the aim of predicting a protein’s melting temperature due to inserted point mutations.

Besides a practical assessment of ProLaTherm in a lab setup, further evaluating the influencing factors for its predictions is important for future research, as this could lead to indications for artificially engineered proteins. Existing explainability approaches for transformer-based models such as attention rollout provide information on the importance of each amino acid in a sequence, i.e. the primary structure (67). However, as outlined, secondary and tertiary structures contain important information regarding thermostability. Thus, connecting the attention rollout scores on the primary structure with secondary and tertiary information is highly relevant for future research.

## Conclusion

In this paper, we present ProLaTherm, a novel protein language model-based thermophilicity prediction model. We benchmark ProLaTherm against feature-, sequence- and literature-based comparison partners on a newly generated dataset. Our experiments show that ProLaTherm outperforms existing methods on several evaluation metrics, both in a nested cross-validation setup and on a test set containing species that do not occur in training. With respect to MCC, ProLaTherm surpasses the second-best competitor by 18.1% in a nested cross-validation setup. Using proteins from species not overlapping with species in the training data, the protein language model-based method outperforms all competitors by at least 9.7%. Furthermore, we detect an even further improved prediction performance with a false negative rate of 2.6% for proteins with an OGT above 70°C. Hence, embeddings from pretrained protein language models seem to be beneficial for this prediction task.

## Supplementary Material

lqad087_Supplemental_File

## Data Availability

All data, including meta-information, and the entire code to reproduce our study and apply ProLaTherm are publicly available at GitHub: https://github.com/grimmlab/ProLaTherm. In addition, the repository version to reproduce this study can be found at Zenodo: https://doi.org/10.5281/zenodo.8354167.
