# Supplementary material for: Superior protein thermophilicity prediction with protein language model embeddings
Source: NAR Genom Bioinform. 2023 Oct 11;5(4):lqad087. doi: 10.1093/nargab/lqad087 (PMC10566323; doi:10.1093/nargab/lqad087)
Supplement: lqad087_Supplemental_File [file lqad087_supplemental_file.pdf]

Published online DD MM YYYY *NAR Genomics and Bioinformatics, 2023, Vol. xx, No. xx* **1–12**
doi:10.1093/nar/nargab000

Superior Protein Thermophilicity Prediction With Protein Language Model Embeddings

Supplementary Materials

Florian Haselbeck [1,2], Maura John [1,2], Yuqi Zhang [1], Jonathan Pirnay [1,2], Juan Pablo Fuenzalida-Werner [3], Rubén D. Costa [3] and Dominik G. Grimm [1,2,4,*]

[1]Technical University of Munich, Campus Straubing for Biotechnology and Sustainability, Bioinformatics, 94315 Straubing, Germany, [2]Weihenstephan-Triesdorf University of Applied Sciences, Bioinformatics, 94315 Straubing, Germany, [3]Technical University of Munich, Campus Straubing for Biotechnology and Sustainability, Chair of Biogenic Functional Materials, 94315 Straubing, Germany, [4]Technical University of Munich, TUM School of Computation, Information and Technology (CIT), 85748 Garching, Germany
[*]Corresponding author: dominik.grimm@hswt.de

Received YYYY-MM-DD; Revised YYYY-MM-DD; Accepted YYYY-MM-DD

SUPPLEMENTARY METHODS

Basic Descriptors

The *weight* descriptor is defined as the difference of the sum of the molecular weight of each amino acid in the protein sequence and the molecular weight of a water molecule for each peptide bound in grams per mol. The *charge* descriptor consists of three features: the net charge, the number of amino acids with positively charged side chains and the number of amino acids carrying negatively charged side chains. Here the amino acids H, K and R are specified to carry positive charges; D and E to carry negative charges; and the remaining 15 are defined as uncharged. For *polarity* we used the number of amino acids with polar side chains and the number of amino acids with non-polar side chains as features, where A, F, G, I, L, M, P, V and W were considered to be non-polar and the remaining 11 residues to be polar. Additionally, we considered the number of *aromatic* amino acids (F, W, Y) in a peptide. For the *mean hydrophobicity* of a peptide we used the hydrophobicity scale PRAM900101 (1). The *mean vdW volume* is defined to be the sum of the normalized van der Waals volume of the residues averaged over the number of amino acids in the peptide.

Amino Acid Composition

The *amino acid composition* (AAC) describes the frequencies of each of the 20 residues in a protein sequence. Thus, for a protein sequence ρ and an amino acid $a\in\{A,C,D,E,F,G,H,I,K,L,M,N,P,Q,R,S,T,V,W,Y\}$ the AAC feature is given as

$$\mathrm{AAC}(\rho,a)=\frac{l(a)}{l_\rho}\in\mathbb{R} \tag{1}$$

where l_ρ is the length of the sequence ρ and $l(a)$ denotes the number of residues of type a in ρ.

Dipeptide Composition

The *dipeptide composition* (DPC) measures the frequencies of each of the 400 possible contiguous dipeptides in a protein sequence. Hence, for a protein sequence ρ and two amino acids $a,b\in\{A,C,D,E,F,G,H,I,K,L,M,N,P,Q,R,S,T,V,W,Y\}$ the DPC feature is defined as

$$\mathrm{DPC}(\rho,(a,b))=\frac{l(a,b)}{l_\rho-1}\in\mathbb{R} \tag{2}$$

where l_ρ denotes again the length of ρ and $l(a,b)$ is the number of contiguous dipeptides of type (a,b) in ρ.

Physicochemical Composition

These features describe the distribution patterns of specific physicochemical and structural properties along the protein sequence. We considered seven of those properties: hydrophobicity, where we use the scale PRAM900101 (1), normalized van der Waals

© 2023 The Author(s)
This is an Open Access article distributed under the terms of the Creative Commons Attribution Non-Commercial License (http://creativecommons.org/licenses/by-nc/2.0/uk/) which permits unrestricted non-commercial use, distribution, and reproduction in any medium, provided the original work is properly cited.

volume, polarity, polarizability, charge, secondary structure and solvent accessibility. For each of these properties, the 20 amino acids are distributed among three categories based on (2, 3). The different groups are listed in Table S1.

Table S1. Distribution of amino acids among different physicochemical properties: The first column gives the seven different physicochemical attributes. In the remaining three columns the amino acids are given in parenthesis, sorted into different groups based on their properties.

Attribute	Categories		
Hydrophobicity	Polar (RKEDQN)	Neutral (GASTPHY)	Hydrophobicity (CLVIMFW)
Normalized vdW Volume	0 - 2.78 (GASCTPD)	2.95 - 4.0 (NVEQIL)	4.43 - 8.08 (MHKFRYW)
Polarity	4.9 - 6.2 (LIFWCMVY)	8.0 - 9.2 (PATGS)	10.4 - 13.0 (HQRKNED)
Polarizability	0 - 0.108 (GASDT)	0.128 - 0.186 (CPNVEQIL)	0.219 - 0.409 (KMHFRYW)
Charge	Positive (KR)	Neutral (ANCQGHILMFPSTWYV)	Negative (DE)
Secondary Structure	Helix (EALMQKRH)	Strand (VIYCWFT)	Coil (GNPSD)
Solvent Accessibility	Buried (ALFCGIVW)	Exposed (PKQEND)	Intermediate (MPSTHY)

Composition The *composition* (CTDC) descriptor measures for each property and each category in Table S1 the corresponding fraction of residues in the sequence, leading to 21 features in total. Hence, for a protein sequence ρ, a physicochemical property q and a corresponding group r the CTDC feature is defined as

$$\mathrm{CTDC}(\rho,q,r) = \frac{l(r\,|\,q)}{l_\rho} \in \mathbb{R} \tag{3}$$

where $l(r\,|\,q)$ denotes the number of residues of group r and property q in the sequence.

Transition The *transition* (CTDT) descriptor measures for each property in Table S1 the fraction of dipeptides in the sequence where the two contiguous amino acids belong to two different groups, e.g. where a residue of group 1 is followed by a residue of group 2 or the other way round. This leads to 21 features in total. For a protein sequence ρ, a physicochemical property q and two corresponding groups r and s the CTDT feature is defined as

$$\mathrm{CTDT}(\rho,q,\{r,s\}) = \frac{l(\{r,s\}\,|\,q)}{l_\rho - 1} \in \mathbb{R} \tag{4}$$

where $l(\{r,s\}\,|\,q)$ is the number of dipeptides (a,b) in ρ, where either a belongs in group r and b belongs in group s or a belongs in group s and b belongs in group r.

Distribution The *distribution* (CTDD) descriptor consists of five values for each property and each category in Table S1, leading to 105 features in total. These values are given by the fractions of the entire sequence where the first amino acid of the corresponding group is located and where 25 %, 50 %, 75 % and 100 % of residues within a group are contained. For a protein sequence ρ, a physicochemical property q, a corresponding group r and a characteristic c (corresponding to the first amino acid or to 25 %, 50 %, 75 % or 100 % of residues) the CTDD feature is defined as

$$\mathrm{CTDD}(\rho,q,r,c) = \frac{i^c_{(r|q)}}{l_\rho} \in \mathbb{R} \tag{5}$$

with $i^c_{(r|q)}$ denoting the position in the protein sequence where the portion of residues of property q and group r corresponding to c occurred.

Pseudo Amino Acid Composition

The *pseudo amino acid composition* (PAAC) includes information on the amino acid composition as well as additional discrete values that reflect the sequence order effect (4). Given a protein sequence ρ of length l_ρ, let a_i denote the ith amino acid in ρ for $i \in \{1,\ldots,l_\rho\}$. Furthermore, denote by $\vartheta_1(a_i)$, $\vartheta_2(a_i)$ and $\vartheta_3(a_i)$ the hydrophobicity value, hydrophilicity value and side-chain mass of a_i after a standard conversion as described in (4), respectively. Define the correlation function as

$$\Psi(a_i,a_{i'}) = \frac{1}{3}\Big((\vartheta_1(a_{i'}) - \vartheta_1(a_i))^2 + (\vartheta_2(a_{i'}) - \vartheta_2(a_i))^2 + (\vartheta_3(a_{i'}) - \vartheta_3(a_i))^2\Big) \tag{6}$$

Then for $\lambda \in \mathbb{N}$ with $\lambda < l_p$, the sequence order effect can be approximated via the following correlation factors:

$$\tau_1 = \frac{1}{l_\rho - 1} \sum_{i=1}^{l_\rho - 1} \Psi(a_i, a_{i+1}) \tag{7}$$

$$\tau_2 = \frac{1}{l_\rho - 2} \sum_{i=1}^{l_\rho - 2} \Psi(a_i, a_{i+2}) \tag{8}$$

$$\vdots \tag{9}$$

$$\tau_\lambda = \frac{1}{l_\rho - \lambda} \sum_{i=1}^{l_\rho - \lambda} \Psi(a_i, a_{i+\lambda}) \tag{10}$$

$$\tag{11}$$

where τ_k is called the kth-tier correlation factor for $k \in \{1,\ldots,\lambda\}$. Now, without loss of generality, denote the 20 amino acids by their index $j \in \{1,\ldots,20\}$, when sorted alphabetically according to their single-letter codes. Then the PAAC features are defined as

$$\text{PAAC}(\rho) = (\rho_u)_{u \in \{1,\ldots,20+\lambda\}} \in \mathbb{R}^{20+\lambda} \tag{12}$$

with

$$\rho_u = \begin{cases} \frac{f_u}{\sum_{j=1}^{20} f_j + \omega \sum_{k=1}^{\lambda} \tau_k}, & \text{if } 1 \leq u \leq 20 \\ \frac{\omega \tau_{u-20}}{\sum_{j=1}^{20} f_j + \omega \sum_{k=1}^{\lambda} \tau_k}, & \text{if } 20+1 \leq u \leq 20+\lambda \end{cases} \tag{13}$$

where f_j for $j \in \{1,\ldots,20\}$, denotes the normalized occurrence frequency of amino acid j in the peptide ρ and ω is a weight factor. In our study we used $\omega = 0.05$ and $\lambda = 3$.

SUPPLEMENTARY TABLES

Table S2. List of thermophilic species and number of proteins in our full dataset before filtering for sequence length and CD-HIT. Species marked with an asterisk were used for our independent test set.

Species	Number of proteins	Species	Number of proteins
Acetivibrio thermocellus	33	*Pyrococcus woesei*	1
Acidianus ambivalens *	12	*Rhodothermus marinus* *	9
Aciduliprofundum boonei *	1	*Rubrobacter xylanophilus*	2
Aeropyrum pernix	50	*Saccharolobus solfataricus*	195
Alicyclobacillus acidocaldarius	5	*Spirochaeta thermophila* *	2
Aquifex aeolicus	111	*Staphylothermus marinus*	2
Aquifex pyrophilus *	3	*Sulfolobus acidocaldarius*	87
Archaeoglobus fulgidus	154	*Sulfolobus islandicus*	6
Archaeoglobus profundus *	8	*Sulfophobococcus zilligii* *	1
Caldalkalibacillus thermarum *	1	*Sulfurisphaera tokodaii*	52
Caldanaerobacter subterraneus	6	*Symbiobacterium thermophilum*	3
Caldicellulosiruptor saccharolyticus *	2	*Thermoanaerobacter ethanolicus* *	1
Carboxydothermus hydrogenoformans	4	*Thermoanaerobacter italicus* *	1
Deferribacter desulfuricans *	1	*Thermoanaerobacter kivui*	1
Dictyoglomus thermophilum	3	Thermoanaerobacterium saccharolyticum *	4
Fervidobacterium pennivorans *	1	*Thermoanaerobacterium thermosulfurigenes* *	4
Geobacillus kaustophilus *	38	*Thermococcus barophilus* *	1
Geobacillus thermodenitrificans	11	*Thermococcus cleftensis* *	2
Geoglobus acetivorans *	1	*Thermococcus fumicolans* *	2
Hydrogenobacter thermophilus *	9	*Thermococcus gammatolerans* *	1
Hyperthermus butylicus *	3	*Thermococcus gorgonarius*	1
Ignicoccus hospitalis *	5	*Thermococcus hydrothermalis*	1
Ignisphaera aggregans *	1	*Thermococcus kodakarensis* *	138
Meiothermus ruber *	1	*Thermococcus litoralis* *	19
Metallosphaera cuprina	1	*Thermococcus onnurineus* *	6
Metallosphaera prunae	1	*Thermococcus profundus* *	1
Metallosphaera sedula *	12	*Thermococcus zilligii* *	2
Methanocaldococcus fervens *	1	*Thermodesulfobacterium geofontis* *	1
Methanocaldococcus infernus *	2	*Thermoleophilum album*	1
Methanocaldococcus jannaschii	315	*Thermoplasma acidophilum*	31
Methanopyrus kandleri	16	*Thermoplasma volcanium*	13
Methanothermobacter marburgensis	59	*Thermoproteus tenax* *	19
Methanothermobacter thermautotrophicus	117	*Thermosediminibacter oceani* *	1
Methanothermococcus thermolithotrophicus *	6	*Thermosipho africanus*	2
Methanothermus fervidus *	8	*Thermosulfidibacter takaii* *	2
Methanotorris igneus *	2	*Thermotoga maritima*	260
Nanoarchaeum equitans	3	*Thermotoga neapolitana*	10
Parageobacillus thermoglucosidasius *	1	*Thermotoga petrophila*	2
Persephonella marina *	2	*Thermovibrio ammonificans* *	1
Picrophilus torridus *	10	*Thermus aquaticus* *	25
Pyrobaculum aerophilum	21	*Thermus filiformis* *	2
Pyrobaculum calidifontis *	10	*Thermus scotoductus* *	3
Pyrobaculum islandicum	3	*Thermus thermophilus*	455
Pyrococcus abyssi	75	*Ureibacillus thermosphaericus* *	1
Pyrococcus furiosus	213	*Vulcanisaeta distributa* *	1
Pyrococcus horikoshii	147		

Table S3. List of non-thermophilic species and number of proteins in our full dataset before filtering for sequence length and CD-HIT. Species marked with an asterisk were used for our independent test set.

Species	Number of proteins	Species	Number of proteins
Acaryochloris marina	3	*Formosa agariphila* ∗	36
Acidithiobacillus ferridurans ∗	2	*Geobacter metallireducens*	3
Acidithiobacillus thiooxidans ∗	2	*Geobacter sulfurreducens*	10
Actinoplanes missouriensis ∗	3	*Gillisia limnaea* ∗	1
Aeromonas hydrophila	11	*Gluconacetobacter diazotrophicus*	2
Agrobacterium fabrum	52	*Gluconobacter oxydans*	9
Agrobacterium radiobacter	5	*Gluconobacter thailandicus*	2
Agrobacterium tumefaciens	5	*Halalkalibacterium halodurans*	20
Albidiferax ferrireducens	1	*Haliscomenobacter hydrossis* ∗	1
Alcanivorax borkumensis ∗	4	*Halobacillus andaensis* ∗	1
Aliivibrio fischeri	10	*Halomonas halodenitrificans* ∗	3
Aquaspirillum arcticum ∗	1	*Halothiobacillus neapolitanus* ∗	15
Aquincola tertiaricarbonis ∗	4	*Herminiimonas arsenicoxydans*	3
Aromatoleum aromaticum	4	*Hyphomicrobium methylovorum* ∗	3
Asticcacaulis excentricus ∗	4	*Hyphomicrobium sulfonivorans* ∗	1
Bacillus atrophaeus ∗	3	*Ideonella sakaiensis* ∗	2
Bacillus mojavensis	3	*Ilyobacter polytropus* ∗	2
Bacillus subtilis	1951	*Lacinutrix mariniflava* ∗	1
Bartonella bacilliformis	1	*Lactococcus lactis*	75
Bradyrhizobium diazoefficiens	17	*Leifsonia aquatica* ∗	1
Brevibacillus laterosporus	1	*Leptospira interrogans*	10
Carnobacterium maltaromaticum ∗	7	*Leuconostoc mesenteroides*	15
Catenulispora acidiphila ∗	2	*Leucothrix mucor*	1
Cellvibrio japonicus	17	*Lewinella persica* ∗	1
Chitinophaga pinensis ∗	3	*Lysobacter antibioticus* ∗	3
Chlamydia trachomatis	41	*Marivirga tractuosa* ∗	1
Chondromyces crocatus ∗	2	*Martelella endophytica* ∗	1
Chromobacterium violaceum	10	*Mesorhizobium japonicum* ∗	16
Clavibacter michiganensis	1	*Methanococcoides burtonii* ∗	3
Clostridium botulinum	15	*Methanococcus vannielii*	6
Colwellia psychrerythraea	5	*Methanosphaerula palustris* ∗	1
Corynebacterium ammoniagenes ∗	2	*Methylorubrum extorquens*	20
Cupriavidus metallidurans	22	*Moritella abyssi* ∗	2
Cupriavidus necator	40	*Moritella profunda* ∗	1
Cupriavidus pinatubonensis	5	*Mycoplasma genitalium*	7
Cyclobacterium marinum ∗	1	*Mycoplasmopsis agalactiae*	1
Cytophaga hutchinsonii	2	*Myxococcus fulvus* ∗	2
Dehalococcoides mccartyi ∗	6	*Myxococcus xanthus*	45
Deinococcus radiodurans	70	*Nonlabens dokdonensis* ∗	1
Delftia acidovorans	6	*Nonlabens ulvanivorans* ∗	4
Desulfotalea psychrophila ∗	1	*Oceanicola granulosus* ∗	2
Escherichia coli	259	*Oceanobacillus iheyensis*	1
Flavobacterium frigidimaris ∗	1	*Oenococcus oeni*	3
Flavobacterium johnsoniae	6	*Paenarthrobacter aurescens*	1
Flavobacterium psychrophilum	1	*Paenibacillus amylolyticus* ∗	1

Table S4. List of non-thermophilic species and number of proteins in our full dataset before filtering for sequence length and CD-HIT. Species marked with an asterisk were used for our independent test set.

Species	Number of proteins	Species	Number of proteins
Paenibacillus lemnae ∗	1	*Saccharopolyspora erythraea*	19
Paenibacillus thiaminolyticus ∗	1	*Salinispora arenicola* ∗	1
Paludibacter propionicigenes	1	*Salinispora tropica*	4
Paraburkholderia phytofirmans	1	*Shewanella colwelliana*	1
Paraburkholderia xenovorans	9	*Shewanella frigidimarina*	3
Paracoccus denitrificans	48	*Shewanella halifaxensis*	1
Pectobacterium atrosepticum	17	*Shewanella oneidensis* ∗	34
Pelagibacterium halotolerans ∗	1	*Shewanella pealeana* ∗	1
Photobacterium phosphoreum ∗	7	*Shigella flexneri*	55
Photobacterium profundum	3	*Singulisphaera acidiphila* ∗	1
Polaromonas naphthalenivorans	1	*Sinorhizobium medicae*	2
Polaromonas sp.	3	*Sodalis glossinidius*	1
Prosthecochloris aestuarii	3	*Sorangium cellulosum* ∗	4
Pseudoalteromonas atlantica ∗	5	*Sphingosinicella xenopeptidilytica* ∗	1
Pseudoalteromonas carrageenovora ∗	2	*Staphylococcus xylosus* ∗	7
Pseudoalteromonas haloplanktis	4	*Starkeya novella* ∗	7
Pseudoalteromonas piscicida ∗	2	*Stigmatella aurantiaca* ∗	7
Pseudoalteromonas translucida	3	*Streptomyces avermitilis*	16
Pseudomonas aeruginosa	424	*Streptomyces cyslabdanicus* ∗	2
Pseudomonas entomophila	3	*Streptomyces muensis* ∗	1
Pseudomonas fluorescens	37	*Streptomyces tsukubensis* ∗	1
Pseudomonas marginalis ∗	3	*Sulfurimonas autotrophica* ∗	1
Pseudooceanicola batsensis ∗	1	*Sulfurospirillum multivorans* ∗	2
Psychrobacter arcticus	3	*Synechocystis sp.*	142
Psychrobacter cryohalolentis	2	*Thalassotalea agarivorans*	1
Psychrobacter immobilis ∗	2	*Thiothrix nivea* ∗	1
Psychroflexus torquis ∗	1	*Vibrio campbellii*	3
Psychromonas ingrahamii	2	*Vibrio cholerae serotype*	147
Renibacterium salmoninarum	1	*Vibrio harveyi*	13
Rhizobium leguminosarum	24	*Vibrio metoecus* ∗	1
Rhizobium meliloti	54	*Vibrio parahaemolyticus*	26
Rhizobium radiobacter	28	*Vibrio vulnificus*	14
Rhodococcus erythropolis	23	*Xanthomonas axonopodis*	3
Rhodopirellula baltica	1	*Xanthomonas campestris*	40
Rhodopseudomonas palustris	81	*Xanthomonas citri*	3
Rhodospirillum rubrum	27	*Xylella fastidiosa*	9
Rickettsia prowazekii	11	*Yersinia enterocolitica*	47
Roseivirga ehrenbergii ∗	2	*Yersinia pestis*	77
Roseovarius nubinhibens ∗	3	*Yersinia pseudotuberculosis*	25
Runella zeae ∗	2	*Zunongwangia profunda* ∗	1
Saccharibacillus brassicae ∗	1	*Zymomonas mobilis*	19
Saccharophagus degradans	1		

Table S5. Hyperparameters and ranges optimized for `ProLaTherm`: Numbers reflect a list of potential values (curly brackets) or the lower respective upper bound (square brackets), with a step size of 1 for integer values as default. In some cases, specific step sizes Δ were used instead of a continuous search space.

Hyperparameter	Values	Notes
	**ProLaTherm**	
dropout	$[0,0.5]$ with $\Delta=0.1$	dropout rate for dropout layers
learning_rate	$\{10^{-5},10^{-4},10^{-3},10^{-2},10^{-1}\}$	learning rate of the Adam optimizer
early_stopping_patience	$[0,10]$ with $\Delta=5$	epochs without improvement needed for early stopping
batch_size	$\{64,128,256\}$	batch size for training
n_init_units_factor	$[0.5,1]$ with $\Delta=0.05$	number of neurons in the first fully-connected layer in relation to the number of input features

Table S6. Hyperparameters and ranges optimized for the feature-based comparison partners Elastic Net, SVM, Random Forest, XGBoost and MLP: Numbers reflect a list of potential values (curly brackets) or the lower respective upper bound (square brackets), with a step size of 1 for integer values as default. In some cases, specific step sizes Δ were used instead of a continuous search space.

Hyperparameter	Values	Notes
	**Elastic Net**	
C	$[10^{-3},10^{3}]$	weighting factor of the regularization terms
l1_ratio	$[0.05,0.95]$ with $\Delta=0.05$	trade off between L1- and L2-regularization
	**SVM**	
kernel	{'linear', 'poly', 'rbf'}	kernel function to use
C	$[10^{-3},10^{3}]$	regularization factor
degree	$[1,5]$	polynomial degree of kernel function (*if kernel is 'poly'*)
gamma	$[10^{-3},10^{3}]$	kernel coefficient (*if kernel is 'rbf' or 'poly'*)
	**Random Forest**	
n_estimators	$\{50,100,250,500,750,1000,1250,1500,1750,2000,$ $2250,2500,2750,3000,3500,4000,4500,5000\}$	number of trees in the ensemble
min_samples_split	$[0.005,0.9]$ with $\Delta=0.005$	minimum ratio of the number of samples to split a node
max_depth	$[2,50]$ with $\Delta=2$	maximum depth of a tree
min_samples_leaf	$[0.005,0.5]$ with $\Delta=0.005$	minimum ratio of the number of samples at a leaf node
max_features	{'sqrt', 'log2'}	number of features to consider at determining best split
	**XGBoost**	
n_estimators	$\{50,100,250,500,750,1000,1250,1500,$ $1750,2000,2250,2500,2750,3000\}$	number of trees in the ensemble
max_depth	$[2,20]$	maximum depth of a tree
learning_rate	$[0.025,0.5]$ with $\Delta=0.025$	boosting learning rate
gamma	$[0,10]$ with $\Delta=0.1$	minimum loss reduction for a further partition on a leaf node
subsample	$[0.05,0.95]$ with $\Delta=0.05$	subsample ratio of training instances for tree construction
colsample_bytree	$[0.05,0.95]$ with $\Delta=0.05$	ratio of features to use for each tree
reg_alpha	$[0,10]$ with $\Delta=0.1$	L1-regularization term on weights
	**MLP**	
dropout	$[0,0.5]$ with $\Delta=0.1$	dropout rate for dropout layers
act_function	{'relu', 'tanh'}	activation function to use
learning_rate	$\{10^{-5},10^{-4},10^{-3},10^{-2},10^{-1}\}$	learning rate of the Adam optimizer
early_stopping_patience	$[0,10]$ with $\Delta=5$	epochs without improvement needed for early stopping
batch_size	$\{64,128,256\}$	batch size for training
n_layers	$[1,5]$	number of blocks consisting of a fully-connected, batch normalization and dropout layer
n_init_units_factor	$[0.1,0.95]$ with $\Delta=0.05$	number of neurons in the first fully-connected layer in relation to the number of input features
perc_dec	$[0.1,0.5]$ with $\Delta=0.05$	percentage decrease of number of neurons per building block

Table S7. Hyperparameters and ranges optimized for the hybrid sequence-based comparison partners LSTM_BasicDesc and Bi-LSTM_BasicDesc as well as the purely sequence-based models MLP_Embedding, LSTM and Bi-LSTM: Numbers reflect a list of potential values (curly brackets) or the lower respective upper bound (square brackets), with a step size of 1 for integer values as default. In some cases, specific step sizes Δ were used instead of a continuous search space.

Hyperparameter	Values	Notes
	**LSTM_BasicDesc**	
dropout	$[0,0.5]$ with $\Delta = 0.1$	dropout rate for dropout layers
act_function	{'relu', 'tanh'}	activation function to use
learning_rate	$\{10^{-5}, 10^{-4}, 10^{-3}, 10^{-2}, 10^{-1}\}$	learning rate of the Adam optimizer
early_stopping_patience	$[0,10]$ with $\Delta = 5$	epochs without improvement needed for early stopping
batch_size	$\{64, 128, 256\}$	batch size for training
n_lstm_layers	[1, 3]	number of LSTM layers
hidden_size_exp	[3, 8]	dimensionality of hidden states as exponent with base 2
n_init_units_factor	$[0.5,1]$ with $\Delta = 0.05$	number of neurons in the first fully-connected layer in relation to the number of input features
latent_dim_exp	[3, 8]	final dimensionality of latent representation after linear layers
n_lin_layer	[0, 3]	number of linear layers to reach latent_dim_exp, with 0 reflecting no dimensionality increase
	**Bi-LSTM_BasicDesc**	
dropout	$[0,0.5]$ with $\Delta = 0.1$	dropout rate for dropout layers
act_function	{'relu', 'tanh'}	activation function to use
learning_rate	$\{10^{-5}, 10^{-4}, 10^{-3}, 10^{-2}, 10^{-1}\}$	learning rate of the Adam optimizer
early_stopping_patience	$[0,10]$ with $\Delta = 5$	epochs without improvement needed for early stopping
batch_size	$\{64, 128, 256\}$	batch size for training
n_lstm_layers	[1, 3]	number of LSTM layers
hidden_size_exp	[3, 8]	dimensionality of hidden states as exponent with base 2
n_init_units_factor	$[0.5,1]$ with $\Delta = 0.05$	number of neurons in the first fully-connected layer in relation to the number of input features
latent_dim_exp	[3, 8]	final dimensionality of latent representation after linear layers
n_lin_layer	[0, 3]	number of linear layers to reach latent_dim_exp, with 0 reflecting no dimensionality increase
	**MLP_Embedding**	
dropout	$[0,0.5]$ with $\Delta = 0.1$	dropout rate for dropout layers
act_function	{'relu', 'tanh'}	activation function to use
learning_rate	$\{10^{-5}, 10^{-4}, 10^{-3}, 10^{-2}, 10^{-1}\}$	learning rate of the Adam optimizer
early_stopping_patience	$[0,10]$ with $\Delta = 5$	epochs without improvement needed for early stopping
batch_size	$\{64, 128, 256\}$	batch size for training
two_layer_head_class	{False, True}	use first fully-connected layer of head classifier architecture
n_init_units_factor	$[0.5,1]$ with $\Delta = 0.05$	number of neurons in the first fully-connected layer in relation to the number of input features
embedding_dim	1024	dimensionality of embedding layer, here set to 1024
	**LSTM**	
dropout	$[0,0.5]$ with $\Delta = 0.1$	dropout rate for dropout layers
act_function	{'relu', 'tanh'}	activation function to use
learning_rate	$\{10^{-5}, 10^{-4}, 10^{-3}, 10^{-2}, 10^{-1}\}$	learning rate of the Adam optimizer
early_stopping_patience	$[0,10]$ with $\Delta = 5$	epochs without improvement needed for early stopping
batch_size	$\{64, 128, 256\}$	batch size for training
n_lstm_layers	[1, 3]	number of LSTM layers
hidden_size_exp	[3, 8]	dimensionality of hidden states as exponent with base 2
n_init_units_factor	$[0.5,1]$ with $\Delta = 0.05$	number of neurons in the first fully-connected layer in relation to the number of input features
embedding_dim	1024	dimensionality of embedding layer, here set to 1024
	**Bi-LSTM**	
dropout	$[0,0.5]$ with $\Delta = 0.1$	dropout rate for dropout layers
act_function	{'relu', 'tanh'}	activation function to use
learning_rate	$\{10^{-5}, 10^{-4}, 10^{-3}, 10^{-2}, 10^{-1}\}$	learning rate of the Adam optimizer
early_stopping_patience	$[0,10]$ with $\Delta = 5$	epochs without improvement needed for early stopping
batch_size	$\{64, 128, 256\}$	batch size for training
n_lstm_layers	[1, 3]	number of LSTM layers
hidden_size_exp	[3, 8]	dimensionality of hidden states as exponent with base 2
n_init_units_factor	$[0.5,1]$ with $\Delta = 0.05$	number of neurons in the first fully-connected layer in relation to the number of input features
embedding_dim_exp	[4, 8]	dimensionality of embedding layer as exponent with base 2

Table S8. Hyperparameters and ranges optimized for the purely sequence-based comparison partners vanilla-Transformer and BigBird: Numbers reflect a list of potential values (curly brackets) or the lower respective upper bound (square brackets), with a step size of 1 for integer values as default. In some cases, specific step sizes Δ were used instead of a continuous search space.

Hyperparameter	Values	Notes
	**vanilla-Transformer**	
dropout	$[0.1, 0.5]$ with $\Delta = 0.1$	dropout rate for dropout layers
learning_rate	$\{10^{-5}, 10^{-4}, 10^{-3}\}$	learning rate of the Adam optimizer
early_stopping_patience	50	epochs without improvement needed for early stopping, set to 50
n_minibatches	{2, 4, 8}	number of minibatches with similar sequence length within each batch of size 1024
embedding_dim_exp	[4, 6]	dimensionality of embeddings as exponent with base 2
kernel_size_avgpool	2, 3, 5	kernel size of average pooling layer
n_heads	[2,6] with $\Delta = 2$	number of heads in each Transformer layer
n_transformer_blocks	[2,6] with $\Delta = 2$	number of Transformer layers
factor_hidden_dim_mlp	[2, 4]	factor of number of neurons in feedforward part of Transformer block in relation to input dimensionality
two_layer_head_class	{False, True}	use first fully-connected layer of head classifier architecture
label_smoothing	{0.0, 0.1}	label smoothing used for loss calculation
weight_decay	$\{10^{-4}, 10^{-3}, 10^{-2}, 10^{-1}\}$	weight decay for Adam optimizer
	**BigBird**	
dropout	$[0.1, 0.5]$ with $\Delta = 0.1$	dropout rate for dropout layers
learning_rate	$\{10^{-5}, 10^{-4}, 10^{-3}\}$	learning rate of the Adam optimizer
early_stopping_patience	50	epochs without improvement needed for early stopping, set to 50
n_minibatches	{4, 8}	number of minibatches with similar sequence length within each batch of size 1024
embedding_dim_exp	[4, 6]	dimensionality of embeddings as exponent with base 2
n_heads	[2,6] with $\Delta = 2$	number of heads in each Transformer layer
n_transformer_blocks	[2,6] with $\Delta = 2$	number of Transformer layers
fact_num_global_tokens	{2,3}	factor of number of global tokens in relation to block size
block_size	{8,16,32}	size of each block for sparse self-attention
two_layer_head_class	{False, True}	use first fully-connected layer of head classifier architecture
label_smoothing	{0.0, 0.1}	label smoothing used for loss calculation
weight_decay	$\{10^{-4}, 10^{-3}, 10^{-2}, 10^{-1}\}$	weight decay for Adam optimizer

Table S9. Overview of the test and validation results with no overlap between the species in the test and cross-validation data: For each prediction model, we show the given evaluation metric on the full test set (*test set 1*) in the first line as well as the mean and standard deviation on the validation sets of the five-fold cross-validation in the second line. The prediction models are grouped as feature-based as well as hybrid and purely sequence-based. With respect to the comparison partners from the literature, we only report the evaluation metrics on the test data as these models were cross-validated using another data split with a potential overlap between the authors' training data and our validation sets. Due to the composition of our test data, we ensure a fair comparison, see Experimental Settings. The best result for each evaluation metric is highlighted in bold - both for the test and validation data. Results for *test set 2* consisting of evolutionary less related proteins can be found in Table 4 of the main paper.

Prediction model	Accuracy	F1-Score	Precision	Recall	Specificity	BACC	MCC
**Feature-based models**							
Elastic Net	0.814<br>(0.911±0.009)	0.822<br>(0.855±0.015)	0.976<br>(0.893±0.015)	0.710<br>(0.821±0.025)	0.973<br>(0.954±0.007)	0.842<br>(0.887±0.013)	0.672<br>(0.793±0.021)
SVM	0.817<br>(0.911±0.009)	0.827<br>(0.858±0.015)	0.969<br>(0.879±0.015)	0.722<br>(0.838±0.019)	0.964<br>(0.946±0.007)	0.843<br>(0.892±0.012)	0.673<br>(0.794±0.022)
Random Forest	0.712<br>(0.872±0.008)	0.695<br>(0.771±0.017)	0.969<br>(0.904±0.016)	0.542<br>(0.673±0.025)	0.973<br>(0.966±0.006)	0.758<br>(0.820±0.012)	0.532<br>(0.700±0.019)
XGBoost	0.840<br>(0.921±0.008)	0.852<br>(0.872±0.014)	0.974<br>(0.905±0.014)	0.757<br>(0.843±0.024)	0.969<br>(0.958±0.007)	0.863<br>(0.900±0.011)	0.710<br>(0.817±0.019)
MLP	0.844<br>(0.915±0.008)	0.856<br>(0.863±0.014)	0.971<br>(0.890±0.005)	0.765<br>(0.839±0.025)	0.964<br>(0.951±0.003)	0.865<br>(0.895±0.012)	0.714<br>(0.803±0.019)
**Hybrid sequence-based models**							
LSTM_BasicDesc	0.837<br>(0.903±0.008)	0.854<br>(0.842±0.015)	0.934<br>(0.877±0.011)	0.786<br>(0.811±0.032)	0.915<br>(0.946±0.007)	0.850<br>(0.878±0.014)	0.685<br>(0.774±0.019)
Bi-LSTM_BasicDesc	0.779<br>(0.908±0.014)	0.781<br>(0.855±0.019)	0.974<br>(0.869±0.042)	0.652<br>(0.842±0.009)	0.973<br>(0.939±0.023)	0.813<br>(0.891±0.010)	0.622<br>(0.788±0.031)
**Purely sequence-based models**							
MLP_Embedding	0.819<br>(0.892±0.016)	0.827<br>(0.829±0.017)	0.984<br>(0.855±0.060)	0.713<br>(0.811±0.040)	0.982<br>(0.931±0.039)	0.848<br>(0.871±0.009)	0.684<br>(0.754±0.029)
LSTM	0.837<br>(0.901±0.008)	0.851<br>(0.842±0.013)	0.953<br>(0.868±0.028)	0.768<br>(0.819±0.025)	0.942<br>(0.940±0.016)	0.855<br>(0.880±0.010)	0.694<br>(0.772±0.019)
Bi-LSTM	0.807<br>(0.909±0.006)	0.818<br>(0.853±0.009)	0.950<br>(0.888±0.033)	0.719<br>(0.823±0.032)	0.942<br>(0.950±0.017)	0.830<br>(0.886±0.009)	0.648<br>(0.790±0.012)
vanilla-Transformer	0.803<br>(0.898±0.007)	0.812<br>(0.833±0.012)	0.964<br>(0.880±0.022)	0.701<br>(0.791±0.019)	0.960<br>(0.948±0.011)	0.831<br>(0.870±0.009)	0.651<br>(0.762±0.018)
BigBird	0.814<br>(0.894±0.009)	0.821<br>(0.825±0.016)	0.984<br>(0.880±0.016)	0.704<br>(0.776±0.021)	0.982<br>(0.950±0.007)	0.843<br>(0.863±0.012)	0.677<br>(0.752±0.022)
`ProLaTherm`	**0.919**<br>(**0.978±0.005**)	**0.929**<br>(**0.966±0.009**)	**0.997**<br>(**0.980±0.005**)	0.870<br>(**0.952±0.015**)	**0.996**<br>(**0.991±0.002**)	**0.933**<br>(**0.971±0.008**)	**0.847**<br>(**0.950±0.012**)
**Comparison partners from literature**							
`ThermoPred` (5)	0.817	0.840	0.895	0.791	0.857	0.824	0.635
`SCMTPP` (6)	0.807	0.821	0.937	0.730	0.924	0.827	0.641
`iThermo` (7)	0.819	0.842	0.893	0.797	0.853	0.825	0.637
`SAPPHIRE` (8)	0.870	0.884	0.966	0.814	0.955	0.885	0.752
`DeepTP`* (9)	0.888	0.903	0.925	**0.882**	0.897	0.889	0.772
`BertThermo` (10)	0.880	0.898	0.931	0.867	0.902	0.884	0.757

* 25 proteins excluded from evaluation metric calculation due to overlap with the comparison partner's training data

Table S10. Mean BLAST identity of thermophilic species from *test set 1*: The total number of proteins per thermophilic organism used in *test set 1* is given, as well as the number of correctly classified proteins. For each species we show the average BLAST sequence identity of the best hit among the thermophilic and non-thermophilic training data.

Species	# proteins	# true positives	BLAST identity thermo.	BLAST identity non-thermo.
Acidianus ambivalens	10	8	0.190	0.114
Aciduliprofundum boonei	1	1	0.568	0.559
Aquifex pyrophilus	3	3	0.348	0.352
Archaeoglobus profundus	8	8	0.480	0.147
Caldalkalibacillus thermarum	1	0	0.265	0.235
Caldicellulosiruptor saccharolyticus	1	1	0.047	0.447
Deferribacter desulfuricans	1	1	0.385	0.424
Fervidobacterium pennivorans	1	1	0.020	0.143
Geobacillus kaustophilus	37	11	0.313	0.499
Geoglobus acetivorans	1	1	0.404	0.323
Hydrogenobacter thermophilus	9	8	0.231	0.239
Hyperthermus butylicus	3	3	0.538	0.222
Ignicoccus hospitalis	5	4	0.493	0.176
Ignisphaera aggregans	1	1	0.540	0.540
Meiothermus ruber	1	0	0.041	0.461
Metallosphaera sedula	10	10	0.490	0.288
Methanocaldococcus fervens	1	1	0.196	0.034
Methanocaldococcus infernus	2	2	0.063	0.094
Methanothermococcus thermolithotrophicus	5	5	0.516	0.275
Methanothermus fervidus	6	6	0.425	0.180
Methanotorris igneus	2	2	0.542	0.073
Parageobacillus thermoglucosidasius	1	1	0.146	0.600
Persephonella marina	2	2	0.195	0.059
Picrophilus torridus	10	6	0.322	0.187
Pyrobaculum calidifontis	10	10	0.271	0.205
Rhodothermus marinus	8	6	0.163	0.179
Spirochaeta thermophila	2	1	0.126	0.189
Sulfophobococcus zilligii	1	1	0.619	0.088
Thermoanaerobacter ethanolicus	1	1	0.301	0.286
Thermoanaerobacter italicus	1	1	0.267	0.534
Thermoanaerobacterium saccharolyticum	3	2	0.334	0.222
Thermoanaerobacterium thermosulfurigenes	3	2	0.297	0.099
Thermococcus barophilus	1	1	0.299	0.304
Thermococcus cleftensis	2	2	0.210	0.132
Thermococcus fumicolans	1	1	0.776	0.115
Thermococcus gammatolerans	1	1	0.831	0.153
Thermococcus kodakarensis	118	118	0.582	0.196
Thermococcus litoralis	16	16	0.519	0.244
Thermococcus onnurineus	5	5	0.489	0.119
Thermococcus profundus	1	1	0.847	0.525
Thermococcus zilligii	1	1	0.534	0.065
Thermodesulfobacterium geofontis	1	1	0.099	0.291
Thermoproteus tenax	18	18	0.280	0.158
Thermosediminibacter oceani	1	1	0.050	0.624
Thermosulfidibacter takaii	2	2	0.275	0.355
Thermovibrio ammonificans	1	1	0.042	0.050
Thermus aquaticus	19	16	0.601	0.329
Thermus filiformis	1	1	0.891	0.028
Thermus scotoductus	3	3	0.394	0.174
Ureibacillus thermosphaericus	1	0	0.647	0.052
Vulcanisaeta distributa	1	1	0.303	0.087

REFERENCES

1. Prabhakaran, M. (1990) The distribution of physical, chemical and conformational properties in signal and nascent peptides. *The Biochemical journal,* **269**(3), 691–696.
2. Tomii, K. and Kanehisa, M. (1996) Analysis of amino acid indices and mutation matrices for sequence comparison and structure prediction of proteins. *Protein engineering,* **9**(1), 27–36.
3. Dubchak, I., Muchnik, I., Mayor, C., Dralyuk, I., and Kim, S.-H. (1999) Recognition of a protein fold in the context of the SCOP classification. *Proteins: Structure, Function, and Bioinformatics,* **35**(4), 401–407.
4. Chou, K. C. (2001) Prediction of protein cellular attributes using pseudo-amino acid composition. *Proteins,* **43**(3), 246–255.
5. Lin, H. and Chen, W. (2011) Prediction of thermophilic proteins using feature selection technique. *Journal of microbiological methods,* **84**(1), 67–70.
6. Charoenkwan, P., Chotpatiwetchkul, W., Lee, V. S., Nantasenamat, C., and Shoombuatong, W. (2021) A novel sequence-based predictor for identifying and characterizing thermophilic proteins using estimated propensity scores of dipeptides. *Scientific reports,* **11**(1), 23782.
7. Ahmed, Z., Zulfiqar, H., Khan, A. A., Gul, I., Dao, F.-Y., Zhang, Z.-Y., Yu, X.-L., and Tang, L. (2022) iThermo: A Sequence-Based Model for Identifying Thermophilic Proteins Using a Multi-Feature Fusion Strategy. *Frontiers in microbiology,* **13**, 790063.
8. Charoenkwan, P., Schaduangrat, N., Moni, M. A., Lio', P., Manavalan, B., and Shoombuatong, W. (2022) SAPPHIRE: A stacking-based ensemble learning framework for accurate prediction of thermophilic proteins. *Computers in biology and medicine,* **146**, 105704.
9. Zhao, J., Yan, W., and Yang, Y. (2023) DeepTP: A Deep Learning Model for Thermophilic Protein Prediction. *International Journal of Molecular Sciences,* **24**(3).
10. Pei, H., Li, J., Ma, S., Jiang, J., Li, M., Zou, Q., and Lv, Z. (2023) Identification of Thermophilic Proteins Based on Sequence-Based Bidirectional Representations from Transformer-Embedding Features. *Applied Sciences,* **13**(5).
